# Sensory reactivity in children and adolescents with autism

**DOI:** 10.1192/j.eurpsy.2024.964

**Published:** 2024-08-27

**Authors:** S. Perez, I. Martin

**Affiliations:** ^1^Psychiatry, Los Arcos del Mar Menor Hospital; ^2^psychiatry, Infante Mental Health Center, Murcia, Spain

## Abstract

**Introduction:**

The gut-brain axis establishes the relationships between bacteria, neurotransmitters and psychophysiological responses associated with a neuronal and behavioral correlate in autism and different mental disorders.

In recent years, there has been an increase in studies on the implications of the gut microbiota (MI) in children with autism spectrum disorders (ASD).

**Objectives:**

1. To study if there is a dysbiosis or alteration of the MI can trigger the appearance of ASD symptoms.

It is considered that there is a frequent comorbidity with gastro-intestinal symptoms (GS), pain and sensory reactivity in ASD, and that these are indicators of a possible alteration in the gut-brain axis.

**Methods:**

In this sense, children with ASD have hypersensitivity to certain visual, olfactory, tactile, etc. stimuli. which makes them be more picky about food and have certain repetitive patterns of behavior, as a consequence they present gastrointestinal symptoms such as constipation and abdominal pain. Sensory reactivity can influence both feeding and sleep patterns in autism.

**Results:**

Currently, there are measuring instruments for sensory reactivity, pain and gastro-intestinal symptoms. However, there are several limitations of these instruments and especially with sensory reactivity in autism because: (1) the items had not been developed in collaboration with interested parties (pediatricians, neuropsychologists, etc.) and (2) the lack of structural validity analysis. Thus, it appears that most validation studies do not meet the criteria of sufficient psychometric quality according to the COSMIN guidelines. Additionally, there is a lack of consensus around terminology (e.g., sensory overreactivity, hyperreactivity, SOR, etc.) and components relevant to sensory functioning.

**Conclusions:**

In the present work, preliminary data are presented on new measures to take into account to evaluate sensory reactivity and pain in the population with autism. This is a first step to obtain an index of the gut-brain axis for the ASD population.

Keywords: ASD, gut-brain, sensory reactivity, pain

**Disclosure of Interest:**

None Declared

